# Neurological Evidence of Diverse Self-Help Breathing Training With Virtual Reality and Biofeedback Assistance: Extensive Exploration Study of Electroencephalography Markers

**DOI:** 10.2196/55478

**Published:** 2024-12-06

**Authors:** Hei-Yin Hydra Ng, Changwei W Wu, Hao-Che Hsu, Chih-Mao Huang, Ai-Ling Hsu, Yi-Ping Chao, Tzyy-Ping Jung, Chun-Hsiang Chuang

**Affiliations:** 1 Research Center for Education and Mind Sciences College of Education National Tsing Hua University Hsinchu Taiwan; 2 Department of Educational Psychology and Counseling College of Education National Tsing Hua University Hsinchu Taiwan; 3 Graduate Institute of Mind, Brain and Consciousness Taipei Medical University New Taipei Taiwan; 4 Research Center of Sleep Medicine Taipei Medical University Hospital Taipei Taiwan; 5 Institute of Computer Science and Engineering National Yang Ming Chiao Tung University Hsinchu Taiwan; 6 Department of Biological Science and Technology National Yang Ming Chiao Tung University Hsinchu Taiwan; 7 Center for Intelligent Drug Systems and Smart Bio-devices (IDS2B) National Yang Ming Chiao Tung University Hsinchu Taiwan; 8 College of Intelligent Computing Chang Gung University Taoyuan Taiwan; 9 Department of Computer Science and Information Engineering Chang Gung University Taoyuan Taiwan; 10 Department of Otorhinolaryngology-Head and Neck Surgery Chang Gung Memorial Hospital Linkou Taiwan; 11 Institute for Neural Computation and Institute of Engineering in Medicine University of California, San Diego La Jolla, CA United States

**Keywords:** biofeedback, virtual reality, breathing training, EEG, electroencephalography, effective connectivity

## Abstract

**Background:**

Recent advancements in virtual reality (VR) and biofeedback (BF) technologies have opened new avenues for breathing training. Breathing training has been suggested as an effective means for mental disorders, but it is difficult to master the technique at the beginning. VR-BF technologies address the problem of breathing, and visualizing breathing may facilitate the learning of breathing training. This study explores the integration of VR and BF to enhance user engagement in self-help breathing training, which is a multifaceted approach encompassing mindful breathing, guided breathing, and breath counting techniques.

**Objective:**

We identified 3 common breathing training techniques in previous studies, namely mindful breathing, guided breathing, and breath counting. Despite the availability of diverse breathing training methods, their varying effectiveness and underlying neurological mechanisms remain insufficiently understood. We investigated using electroencephalography (EEG) indices across multiple breathing training modalities to address this gap.

**Methods:**

Our automated VR-based breathing training environment incorporated real-time EEG, heart rate, and breath signal BF. We examined 4 distinct breathing training conditions (resting, mindful breathing, guided breathing, and breath counting) in a cross-sectional experiment involving 51 healthy young adults, who were recruited through online forum advertisements and billboard posters. In an experimental session, participants practiced resting state and each breathing training technique for 6 minutes. We then compared the neurological differences across the 4 conditions in terms of EEG band power and EEG effective connectivity outflow and inflow with repeated measures ANOVA and paired *t* tests.

**Results:**

The analyses included the data of 51 participants. Notably, EEG band power across the theta, alpha, low-beta, high-beta, and gamma bands varied significantly over the entire scalp (*t* ≥1.96, *P* values <.05). Outflow analysis identified condition-specific variations in the delta, alpha, and gamma bands (*P* values <.05), while inflow analysis revealed significant differences across all frequency bands (*P* values <.05). Connectivity flow analysis highlighted the predominant influence of the right frontal, central, and parietal brain regions in the neurological mechanisms underlying the breathing training techniques.

**Conclusions:**

This study provides neurological evidence supporting the effectiveness of self-help breathing training through the combined use of VR and BF technologies. Our findings suggest the involvement of internal-external attention focus and the dorsal attention network in different breathing training conditions. There is a huge potential for the use of breathing training with VR-BF techniques in terms of clinical settings, the new living style since COVID-19, and the commercial value of introducing VR-BF breathing training into consumer-level digital products. Furthermore, we propose avenues for future research with an emphasis on the exploration of applications and the gamification potential in combined VR and BF breathing training.

**Trial Registration:**

ClinicalTrials.gov NCT06656741; https://clinicaltrials.gov/study/NCT06656741

## Introduction

### Background

Integrated biofeedback (BF) and virtual reality (VR) technologies have been widely applied in recent years [1,2]. Accumulating evidence has shown that the application of integrated BF and VR technologies may mitigate users’ stress levels [3,4], anxiety [4-6], self-reported pain [7,8], and developmental disorders [9,10], and improve people’s cognitive function [11,12]. Previous interventions for psychological disorders have employed a variety of BF modules, including electroencephalography (EEG), electromyography, skin conductance, heart rate, heart rate variability, body temperature, and respiration [13-19]. Integrated VR and BF technologies are especially common for daily self-help training and breathing training [4,20]. For example, VR and BF have been used to promote diaphragmatic breathing [3] and stress regulation with breathing training among police officers [12]. Breathing training with VR and BF technologies shows a promising future in terms of cultivating the mental health of the population, and the understanding of the mechanism underneath breathing training is essential to popularize breathing training among the public [1,3,12].

VR and BF address the challenge of daily training engagement adequately [3,12]. The development of VR is blooming because its interactive nature and gamification elements facilitate self-help training and home-based rehabilitation [21,22]. Self-help training encourages people to engage in self-training in their own time to mitigate illness. Still, a high dropout rate often challenges self-help training because the training is often perceived as boring with no immediate excitement for the participants [4,20,23]. As a new-generation human-computer interface, VR provides an immersive user experience with its 360° visual environment [12,24], thus offering excitement and motivating users to continue training [6,25]. Moreover, VR applications can integrate gaming elements into training, such as storylines, roleplay, progression systems, and rewards [21,22]. VR-BF integration also addresses users’ concerns that training content is boring and meaningless, and thus facilitates training consistency [4,20,23]. Overall, integrated VR and BF interventions provide users with interactive experiences, involvement, and excitement [4,20,23]. For instance, intervention sessions combining VR with BF were developed to improve body coordination and balance in young adults with juvenile idiopathic arthritis [26] and children with cerebral palsy [27], and to improve cognitive function under stressful circumstances in police offices [12]. The fundamental reason behind the effectiveness of VR-BF integrated training is that these technologies allow more effective self-help training by supporting trainees’ cognitive engagement [2,11]. Studies suggested that VR increased people’s engagement with self-help training among both adults and children, as participants reported pleasurable training experiences [11,28,29]. On the other hand, BF (eg, breathe monitoring) increased trainees’ awareness toward their breath, therefore allowing them to regulate their breath if needed [3,23]. BF can further reinforce trainees’ engagement through the use of feedback stimuli, and the gamification design further increases the attractiveness of breathing training [4,23,30,31], as game-like training offers the possibility to facilitate trainees’ immersion and boost engagement [30,32]. Overall, evidence supports that the combined use of VR and BF is a way to increase the engagement of self-help training among trainees.

Clinically, a growing population of therapists has combined VR-BF technologies with breathing training [1,3,33]. Breathing training is a common intervention in self-help training, health care, and mindfulness practice [34,35]. Breathing training interventions have been shown to mitigate stress, pain, anxiety, and depression, and improve relaxation [36-40]. Research has also shown that breathing training can be integrated with breathing signal BF and VR to promote users’ heart function and heart rate variability [1,41]. At the consumer level, many smartphone or smartwatch applications provide different breathing training programs with heart rate or breath rate BF [42,43]. In fact, the quantitative and qualitative feedback from VR and BF breath intervention trainees suggested that VR and BF technologies provide positive, relaxing, and peaceful user experiences [31,44]. Underneath the behavioral benefits of breathing training, review studies suggested that breathing training modulates the vagal tone of the parasympathetic nervous system and thereby increases heart rate variability and respiratory sinus arrhythmia in trainees [39,45,46]. These physiological changes further initiate an autonomic relaxation response and regulate the autonomic nervous system to achieve a balance state [3,47]. Overall, both behavioral and physiological evidence suggests that integrating breathing training with BF and VR is a promising strategy to promote personal health in trainees.

Despite behavioral and physiological evidence, some scientists attempted to study what is going on inside of breath trainees’ brains from the neurological perspective [39,48]. EEG studies on breathing training showed an increase in alpha band power in the prefrontal area during Zen Tanden breathing [49,50] and in the occipital and right temporal areas during mindful breathing [51]. A study also found decreased theta band power in the left frontal, right temporal, and left parietal areas when people practice paced breathing training [52], but other studies reported increased theta band power in the temporal, parietal, central, and occipital areas in mindfulness practitioners and in alternate nostril yoga breathing practitioners [48,51]. Studies also reported increased beta and gamma power in the frontal area and the midline in mindful breathing practitioners, yoga breathing practitioners, and Rinpoche, a group of highly experienced Tibetan Buddhism meditators [53-56], while another study on alternate nostril yoga breathing practitioners showed decreased beta band power [48]. The gamma band effective connectivity also increased in people who received 8 weeks of mindful breathing training [57]. Currently, studies on various breathing training techniques coexist and the neurological evidence of them is inconsistent [11,39,58]. The neurological elaboration of different breathing training techniques is essential to the understanding of the nature of breathing training. Yet, this neurological knowledge gap is unaddressed.

We reviewed previous studies on breathing training and identified 3 basic breath techniques that were mostly employed: mindful breathing, guided breathing, and breath counting [33,59,60]. For example, some mindfulness-based interventions promoted changes in trainees through mindful breathing, emphasizing nonjudgmental awareness of people’s body sensations [59,61]. Another intervention, breath counting, instructed participants to count from 1 to 10, along with their breath [60,62]. Lastly, guided breathing interventions instructed participants to breathe at a certain and slow tempo [33,63]. Although studies supported that these breathing techniques would introduce certain benefits to trainees [33,59-63], the differences in their neurological mechanisms are still largely unknown. We attempted to address this neurological knowledge gap in this study. The understanding of the neurobiological mechanisms underlying different breathing techniques is important for the development of VR-BF breathing training and may unveil our knowledge to human brain neuroscience.

### Study Aim

In this study, we attempted to use EEG to examine the neurobiological mechanism differences of different breathing training techniques in an integrated VR and BF environment. EEG is commonly applied in breathing training studies, as it can easily be integrated with other modalities, such as VR and breath signal sensors [13]. Previous EEG studies on breathing training with VR and BF also demonstrated that EEG suitably works with VR-BF equipment without significant interference [10,64]. The research questions are whether and how people’s neurological mechanisms (indicated by EEG indexes) change when they are performing different breathing training techniques (ie, mindful breathing, guided breathing, and breath counting). This study aimed to examine the EEG indexes systematically, including band power, effective connectivity inflow and outflow, and connectivity flow among different brain regions, and analyze the differences of these indexes when people are practicing different breathing training techniques. This comparison will not only reveal the neurological mechanisms of these techniques, but also verify the feasibility of applying integrated VR and BF techniques for breathing training in healthy populations, like the consumer-level digital products proclaimed (eg, smartphones) [41,65].

## Methods

### Participants

The data were collected between the fall of 2021 and the spring of 2022. The researchers recruited potential participants through online forum advertisements and billboard posters. The inclusion criteria were as follows: age ≥20 years; no previous mindfulness training experience; no history of epilepsy, mental disorder, or neurological illness; and no history of substance abuse (alcohol, drugs, or tobacco). Fifty-three candidates from 2 universities in northern Taiwan who fulfilled the above criteria were included in this study. The data of 2 participants were excluded because of extreme data deviation. Eventually, the analyses included the data of 51 young participants (26 men and 25 women; mean age 22.7 years, SD 2.5 years; range 20-29 years).

### Ethical Considerations

National Yang Ming Chiao Tung University – Research Ethics Center for Human Subject Protection (NYCU-REC) reviewed and approved this study (project number: NCTU-REC-109-037F). All participants provided written informed consent before participation. The participants received New Taiwan Dollar 1000 (about US $32.69) in acknowledgement of their participation. In accordance with the regulations of NYCU-REC, all the research materials and data were secured in the designated location, which was protected from any unauthorized personnel by 3 locks. Only project-related personnel, as listed in NYCU-REC (project number: NCTU-REC-109-037F), could access the data. Only the experiment manipulators and the contact persons between NYCU-REC and the laboratory could access the participants’ personal information. After the experiments were finished, every participant’s identity was replaced by a subject number and deidentified. No material that may identify any of the participants is presented in this paper.

### Experimental Environment

**Figure 1:**
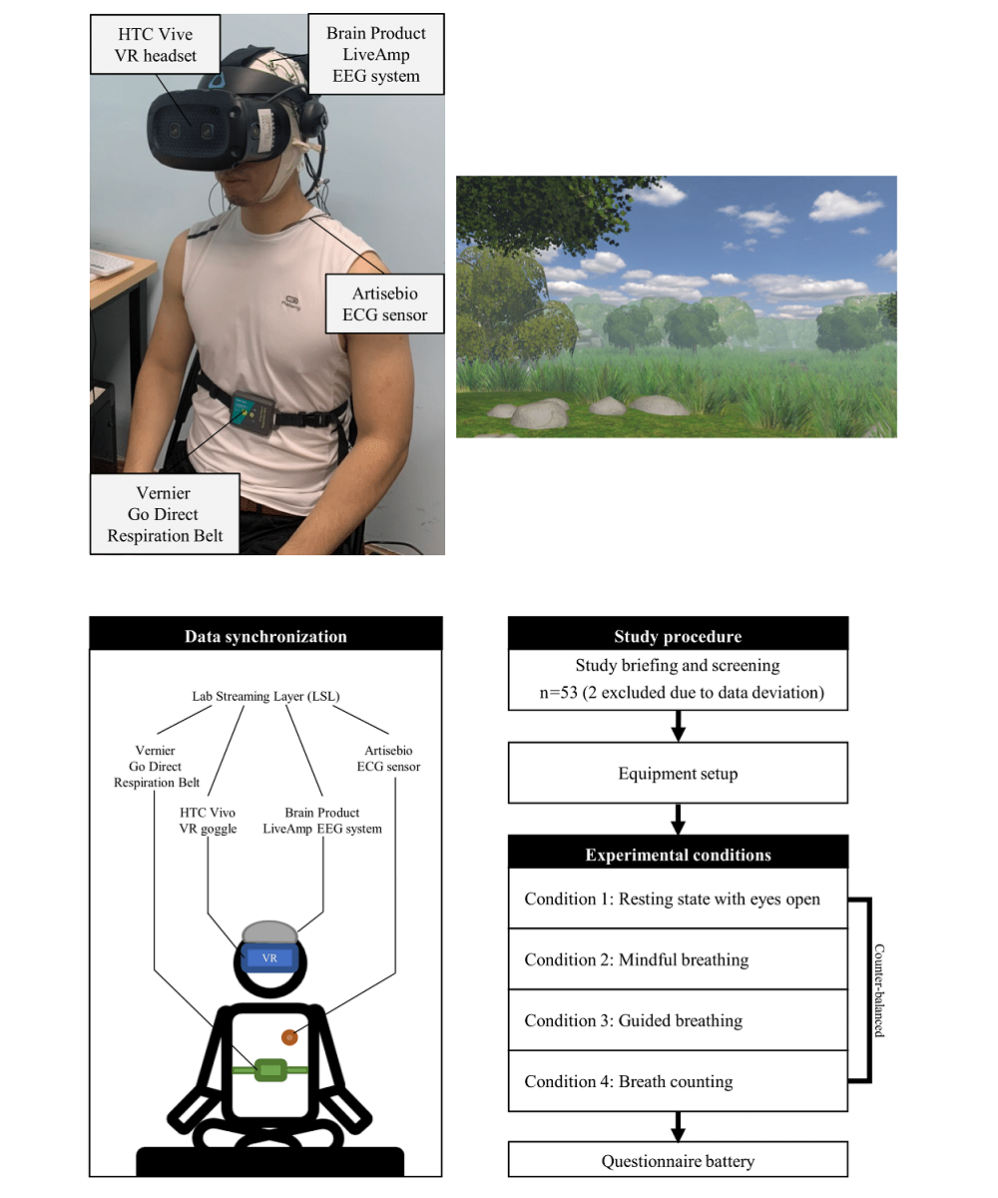
Study procedure and equipment setup. ECG: electrocardiography; EEG: electroencephalography; VR: virtual reality.

The experiment manipulators explained the experimental procedure to the participants and equipped them with an EEG system, an electrocardiography (ECG) sensor, a respiration belt, and a VR headset. The top-left corner of Figure 1 shows the configuration of equipment on a participant during the experiment.

An HTC Vive VR headset was used to create the VR environment, as shown in the top-right corner of Figure 1. This environment featured a tranquil scene of a blue sky and swaying meadow, created using Unity Engine v2019. We adjusted the dynamics of the swaying meadow according to specific experimental conditions.

A 32-channel LiveAmp EEG system (Brain Products GmbH) was used to collect the EEG data at a sampling rate of 1000 Hz and a resolution of 40.7 nV. We mounted the electrodes on EasyCap (Brain Products GmbH), the layout of which followed the international 10/20 electrode placement system [66]. The electrode-skin impedance was maintained under 10 kΩ with an abrasive electrode paste (Abralyt HiCl). EEG data on all channels were acquired using FCz as the reference and were filtered using a band-pass filter of 0.0159 Hz and 250 Hz plus a 60-Hz notch filter.

The ArtiseBio Cygnus-819997-RawEEG system [67] was employed to collect ECG data at a sampling rate of 1000 Hz. Three electrodes from the ArtiseBio system were affixed to Kendall 200 series electrode pads (Cardinal Health) and positioned according to the standard limb leads II configuration for ECG monitoring [68].

A Vernier Go Direct Respiration Belt monitored and captured the respiration data at a sampling rate of 1000 Hz. The respiration belt was worn on the rib cage. After the initiation of each experimental condition (ie, each breathing technique), the program collected 10 seconds of a respiratory signal from each participant for normalization. We compared the respiratory signal with the normalized signal to determine whether the participant was inhaling or exhaling. The participant’s respiration was visualized in the VR environment throughout the experiment, except for the *guided breathing* condition.

The bottom-left corner of Figure 1 presents the synchronized EEG, ECG, and respiratory signals captured during the experiment, which were synchronized and coordinated using the Lab Streaming Layer. Multimedia Appendix 1 shows a screenshot of the synchronized data collection during an experimental session.

### Experimental Procedure

This study was a cross-sectional experiment involving 51 healthy young adults in a single group, and all participants finished all 4 experimental tasks. As such, this study did not have a randomized allocation of participants. The experiment sessions took place in 2 laboratories at 2 universities in northern Taiwan.

We designed the experimental procedure after consultation with 2 certified mindfulness-based stress reduction program instructors. The professional advice suggested that the sensation of breathing may relate to breezing. Therefore, we designed the swaying meadow in a VR environment to visualize a breeze and used the meadow to provide feedback for the participants’ breathing. The participants were new to breathing training, and we did not require them to meet the low respiration rate suggested by the previous mindfulness or breathing training practice guides [34,35,69-71]. The experimental design intentionally instructed the participants to provide their natural response.

The bottom-right corner of Figure 1 provides a visual representation of the experimental procedure. The experiment manipulators briefed the participants on the experimental procedure and assisted them throughout an experimental session in a face-to-face manner. Participants began by spending 5 minutes in a VR environment, immersing themselves in a setting with a tranquil blue sky and a meadow that swayed in response to their respiratory signals. The first 5 minutes was a warm-up session, and no data were recorded yet. Subsequently, the experiment progressed through 4 unique conditions, each lasting 6 minutes. Throughout these conditions, we meticulously recorded EEG, ECG, and respiratory signals. The instruction of each condition was given to the participants verbally by the experiment manipulators before a condition started.

#### Resting State Task (Resting) 

A resting state is a mental state and a research paradigm where people do nothing and idle as much as possible to show their baseline neurological signals [72,73]. In this task, we instructed participants to rest with their eyes open for 6 minutes. The VR environment of a blue sky and swaying meadow was presented to the participants throughout this task.

#### Mindful Breathing Task (Mindful Breathing)

Mindful breathing is a state where people focus on their inner sensation [59,61]. In this task, the meadow’s motion in the VR environment corresponded to the participants’ inhalation and exhalation, as detected by a respiration belt. The participants were instructed to focus mentally on their bodily sensations as they took breaths, with the meadow’s sway as a visual cue.

#### Guided Breathing Task (Guided Breathing)

Guided breathing refers to the breathing training technique where trainees are guided to inhale and exhale according to a fixed tempo [33,63]. In this task, the meadow swayed at a fixed tempo of 4 seconds back and 6 seconds front. The participants were instructed to synchronize their inhalation with the meadow’s backward motion (lasting 4 seconds) and their exhalation with its forward motion (lasting 6 seconds).

#### Breath Counting Task (Breath Counting)

Breath counting refers to the breathing training technique where trainees mentally count the number of breath cycles they have finished in a certain period of time [60,62]. In this task, similar to the mindful breathing task, the meadow’s motion was synchronized with the participants’ breathing patterns. During the 6 minutes, we instructed the participants to breathe naturally and mentally count their breathing cycles.

We counterbalanced the order of experimental tasks to mitigate order effects.

Upon completion of the 4 experimental conditions, a questionnaire package, which included the Five Facet Mindfulness Questionnaire (FFMQ), Montreal Cognitive Assessment (MoCA), Beck Depression Inventory (BDI), Beck Anxiety Inventory (BAI), Mind Wandering: Spontaneous Scale, and Pittsburgh Sleep Quality Index (PSQI), was administered to the participants. These questionnaires were employed based on previous work on breathing training [54,57,74]. Yet, the questionnaire responses were not the focus of this paper. Detailed information and statistics of the questionnaire package can be found in Multimedia Appendix 2.

### Data Analyses

#### EEG Data Preprocessing

All EEG preprocessing procedures were conducted using EEGlab toolbox 2021.1 (Swartz Center for Computational Neuroscience) [75] on MATLAB 2021a. To focus on EEG data that reflected a stable mental state, we excluded the first minute from each 6-minute recording, as implemented in previous EEG studies [54,57]. The EEG dataset underwent an initial down-sampling to a rate of 250 Hz, followed by applying a 1- to 50-Hz band-pass filter employing a Hamming windowed sinc finite impulse response. We used the Artifact Subspace Reconstruction algorithm to correct data bursts exceeding a SD of 20 within a 0.5-second window [76]. Artifacts originating from electrooculograms, electrocardiograms, and electromyograms were identified by independent component analysis in conjunction with ICLabel [77]. Finally, the processed data were rereferenced to the average reference following the established guidelines [78,79].

#### EEG Band Power

Following the procedure of our previous study [54], we converted the preprocessed EEG data to a frequency-domain signal using a short-time Fourier transformation. All transformed spectra were then log-transformed and represented in dB (10log_10_), as Allen et al [78] demonstrated. The spectral data were eventually averaged to calculate the band powers as described in previous work (delta=1-4 Hz; theta=4-8 Hz; alpha=8-12 Hz; low-beta=12-20 Hz; high-beta=20-30 Hz; gamma=30-50 Hz) [80-85]. We calculated the band power using the EEGLAB toolbox 2021.1 (Swartz Center for Computational Neuroscience) [75] with Matlab R2021a.

#### Effective Connectivity Analysis

This study employed the direct directed transfer function (dDTF) to evaluate causal relationships between EEG channels [86]. Modified from the directed transfer function, the dDTF is an effective connectivity estimator grounded in frequency-domain Granger causality [86]. The dDTF isolates and assesses the direct causal link between a specific pair of channels, effectively mitigating the impact of indirect neural influences because of brain tissue conductivity [86].

This study calculated the dDTF using EEGLab Toolbox 2021.1 with the Source Information Flow Toolbox v1.52 plugin [87,88]. Because of computational constraints, we limited our effective connectivity calculations to 19 standard EEG channels (Fp1, Fp2, F3, F4, C3, C4, P3, P4, O1, O2, F7, F8, T7, T8, P7, P8, Fz, Cz, and Pz) out of the available 32 channels.

The EEG effective connectivity values under the 4 conditions were computed separately. All 5-minute-long EEG recordings were segmented into 8-second epochs (ie, 37 epochs per recording) for vector autoregressive (VAR) modeling [89]. Following the implementation in our previous study [57], the VAR models in this study used a sliding-window adaptive modeling approach with a window length of 0.4 seconds and a window step size of 0.05 seconds. We fitted the models using the Vieira-Morf method [90]. The Akaike information criterion (AIC) [91] was used for model order selection. The information in Table 1 confirms the reliability and validity of the parameters used in our Granger causality models, demonstrating satisfactory results for the whiteness, consistency, and stability tests.

**Table 1:** Parameters of vector autoregressive modeling in this study.

Variable^a^			Condition 1: Resting state		Condition 2: Mindful breathing		Condition 3: Guided breathing		Condition 4: Breath counting
Average model order (AIC^b^ suggested)			11.79		11.62		11.77		12.04
**Whiteness tests**									
	Average Ljung-box pass rate	0.959		0.977		0.963		0.913	
	Average autocorrelation function (ACF) pass rate	0.999		0.999		0.999		0.999	
	Average Box-Pierce pass rate	0.965		0.981		0.968		0.925	
	Average LiMcLeod pass rate	0.958		0.975		0.962		0.910	
Average percentage of consistency			66.837		65.408		65.646		65.716
Average stability			1.000		1.000		1.000		1.000

^a^The epoch size was 8 seconds, window length was 0.4 seconds, window step size was 0.05 seconds, and window number per condition was 159 ([8/0.05]-1).

^b^AIC: Akaike information criterion.

This study computed dDTF values for the delta, theta, alpha, low-beta, high-beta, and gamma bands under the definition mentioned above [80-85]. The dDTF values were averaged across windows, yielding a 19×19 matrix of the causal strengths between source and sink channels. This study also calculated the inflow and outflow connectivity from the EEG data. For a given EEG channel, the inflow and outflow connectivity represent the cumulative sum of all corresponding incoming and outgoing connectivity edges, respectively [88].

We grouped EEG channel data into predefined brain regions to better understand connectivity flow among different brain regions, following the method suggested by Djalovski et al [92]. In this study, we identified 6 brain regions: left frontal (Fp1, F3, and F7), right frontal (Fp2, F4, and F8), central (Cz, C3, and C4), parietal (Pz, P3, and P4), left temporal (T7 and P7), and right temporal (T8 and P8). The connectivity magnitude for each brain region was determined by averaging the dDTF values of its respective channels. Analyzing data based on brain regions rather than individual electrodes enhances regional specificity, providing a more meaningful interpretation and a clearer understanding of interregional couplings [93].

### Statistical Analyses

This study used repeated measures ANOVA (rmANOVA) to examine differences in EEG band power for all channels, inflow for sink channels, outflow for source channels, and connectivity patterns among brain regions in the *resting*, *mindful breathing*, *guided breathing*, and *breath counting* conditions. Post-hoc *t* tests were used to further assess pair-wise differences among these conditions. We adjusted all statistical analyses involving multiple comparisons using the false discovery rate (FDR) [94].

## Results

### EEG Band Power Differences Across the 4 Conditions

**Figure 2:**
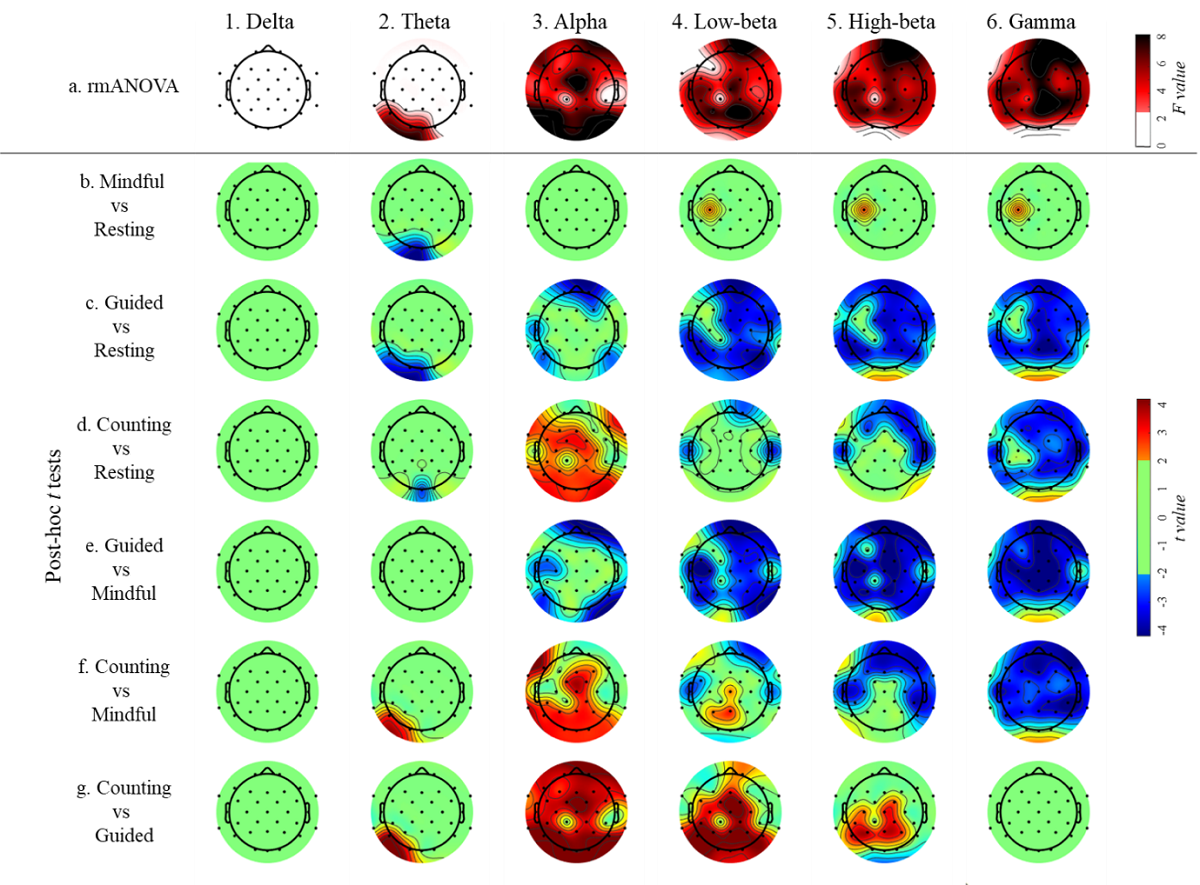
Results of repeated measures ANOVA (rmANOVA) of different band powers across the 4 conditions. False discovery rate (FDR) correction was applied to all the analyses in this figure. All image parts show only the connectivity flow with *P* value with FDR correction (P_FDR_) <0.05, F ≥2.61, and *t* ≥2.01 or ≤–2.01. In all pairwise comparisons, positive t values represent higher mean values of the former condition. Mindful represents mindful breathing, guided represents guided breathing, and counting represents breath counting.

Figure 2 shows the results of the rmANOVA and post-hoc analyses of band powers across the 4 experimental conditions, and Table 2 shows the result summary of EEG band power and EEG effective connectivity outflow and inflow analyses.

For the delta band, no significant differences in band power were detected in any region of the scalp among the 4 experimental conditions (Figure 2 [part 1a]; *P* values >.05).

For the theta band, rmANOVA revealed differences in EEG power specifically in the occipital region (Figure 2 [part 2a]; *P* values <.05). Particularly, post-hoc *t* tests showed that relative to *resting*, the conditions of *mindful breathing*, *guided breathing*, and *breath counting* led to decreased theta power in the occipital region (Figure 2 [parts 2b, 2c, and 2d]; *P* values <.05). Additionally, *breath counting* showed elevated theta power compared with *mindful breathing* and *guided breathing* in the left occipital region (Figure 2 [parts 2f and 2g]; *P* values <.05).

We found significant global band power differences in the alpha, low-beta, high-beta, and gamma frequency bands (Figure 2 [parts 3a, 4a, 5a, and 6a]; *P* values <.05). In the alpha band, *mindful breathing* did not exhibit any band power difference when compared with *resting* (Figure 2 [part 3b]; *P* values >.05). Conversely, *guided breathing* displayed lower band power than *resting* in both the frontal and bilateral parietal regions (Figure 2 [part 3c]; *P* values <.05). In contrast, *breath counting* led to elevated global band power compared with *resting* (Figure 2 [part 3d]; *P* values <.05). *Guided breathing* exhibited lower band power in the frontal, left temporal, and bilateral parietal regions than *mindful breathing* (Figure 2 [part 3e]; *P* values <.05). *Breath counting*, in contrast, demonstrated elevated band power across the entire scalp when compared with both *mindful breathing* and *guided breathing* (Figure 2 [parts 3f and 3g]; *P* values <.05).

**Table 2:** Result summary of electroencephalography band power and electroencephalography effective connectivity outflow and inflow analyses across experimental conditions.

Variable		Resting^a^	Mindful breathing^a^	Guided breathing^a^
**EEG^b^ band power**				
	Mindful breathing	< Theta: occipital > Low-beta: left central > High-beta: left central > Gamma: left central	—^c^	—
	Guided breathing	< Theta: occipital, left temporal < Alpha: frontal, bilateral temporal < Low-beta: global except left frontal < High-beta: global except left central < Gamma: global except left central	< Alpha: frontal, bilateral temporal, right occipital < Low-beta: global except left frontal and occipital < High-beta: global except occipital < Gamma: global except occipital	—
	Breath counting	< Theta: occipital > Alpha: global < Low-beta: right frontal, bilateral temporal < High-beta: frontal, bilateral temporal < Gamma: global except left central	> Theta: left occipital > Alpha: global except right frontal and temporal < Low-beta: right frontal, bilateral temporal > Low-beta: central, parietal < High-beta: frontal, bilateral temporal < Gamma: global except occipital	> Theta: left occipital > Alpha: global except right temporal > Low-beta: global except left frontal and bilateral temporal > High-beta: central, parietal, left temporal
**Effective connectivity outflow**				
	Mindful breathing	—	—	—
	Guided breathing	> Alpha: right frontal, midline central and parietal	> Delta: left parietal > Alpha: right frontal	—
	Breath counting	< Delta: left frontal, left parietal > Alpha: right frontal, right temporal, midline central, left parietal < Gamma: right parietal, occipital	< Delta: left frontal, left parietal > Alpha: right temporal, midline central, left parietal < Gamma: right parietal, occipital	< Delta: left frontal, left parietal > Alpha: bilateral temporal, left parietal < Gamma: right parietal, occipital
**Effective connectivity inflow**				
	Mindful breathing	< Delta: left frontal > Alpha: right frontal > Low-beta: right frontal > High-beta: right frontal > Gamma: left frontal	—	—
	Guided breathing	> Theta: right frontal > Alpha: frontal, right central, midline parietal, left temporal > Low-beta: right frontal > High-beta: right frontal > Gamma: right occipital	> Delta: left occipital > Theta: right frontal > Alpha: right central, midline and right parietal	—
	Breath counting	< Delta: bilateral frontal, right temporal, bilateral parietal, right occipital > Theta: right frontal > Alpha: global < High-beta: midline and left frontal, right central, midline and right parietal, left occipital < Gamma: left frontal, right central, midline parietal	< Delta: bilateral frontal, right temporal, bilateral parietal, right occipital > Alpha: global except left and midline frontal < Low-beta: midline frontal < High-beta: midline and left frontal, right central, midline and right parietal, right temporal, occipital < Gamma: left frontal, midline and right central, midline and left parietal, right temporal, right occipital	< Delta: bilateral frontal, right temporal, bilateral parietal, right occipital > Alpha: bilateral frontal, bilateral temporal, midline central, bilateral parietal, right occipital < Low-beta: midline frontal < High-beta: midline and left frontal, right central, midline and right parietal, right temporal, left occipital < Gamma: left frontal, midline and right central, left parietal, right temporal, right occipital

^a^Symbols “>” and “<” represent the intergroup comparison results of column against row. “>” means the condition in the left column has higher indexes than that of the condition in the top row with *P* values <.05, whereas “<” means the condition in the left column has lower indexes than that of the condition in the top row with *P* values <.05.

^b^EEG: electroencephalography.

^c^Not applicable.

The results of low-beta and high-beta bands were similar (Figure 2 [parts 4a and 5a]). *Mindful breathing* led to elevated band power compared with *resting*, but only in channel C3 (Figure 2 [parts 4b and 5b]; *P* values <.05). *Guided breathing* caused reduced band power across the entire scalp relative to both *mindful breathing* and *resting* (Figure 2 [parts 4c, 4e, 5c, and 5e]; *P* values <.05). *Breath counting* displayed lower band power in the frontal and bilateral regions than *mindful breathing* and *resting* (Figure 2 [parts 4d, 4f, 5d, and 5f]; *P* values <.05). It was noted that *breath counting* showed decreased low-beta band power levels in midline channels (Cz, Pz, and P3) relative to *mindful breathing* (Figure 2 [part 4f]; *P* values <.05), a trend not observed in the high-beta band (Figure 2 [part 5f]; *P* values >.05). Compared with *guided breathing*, *breath counting* exhibited elevated power in both the low-beta and high-beta bands across the central, parietal, occipital, and bilateral temporal regions (Figure 2 [parts 4g and 5g]; *P* values <.05).

For the gamma band, *mindful breathing* showed higher band power than *resting* only in channel C3 (Figure 2 [part 6b]; *P* values <.05). Both *guided breathing* and *breath counting* had lower band power globally than *resting* (Figure 2 [parts 6c and 6d]; *P* values <.05) and *mindful breathing* (Figure 2 [parts 6e and 6f]; *P* values <.05). Lastly, there was no band power difference between *guided breathing* and *breath counting* (Figure 2 [part 6g]; *P* values >.05).

### Connectivity Outflow Differences Across the 4 Conditions

**Figure 3:**
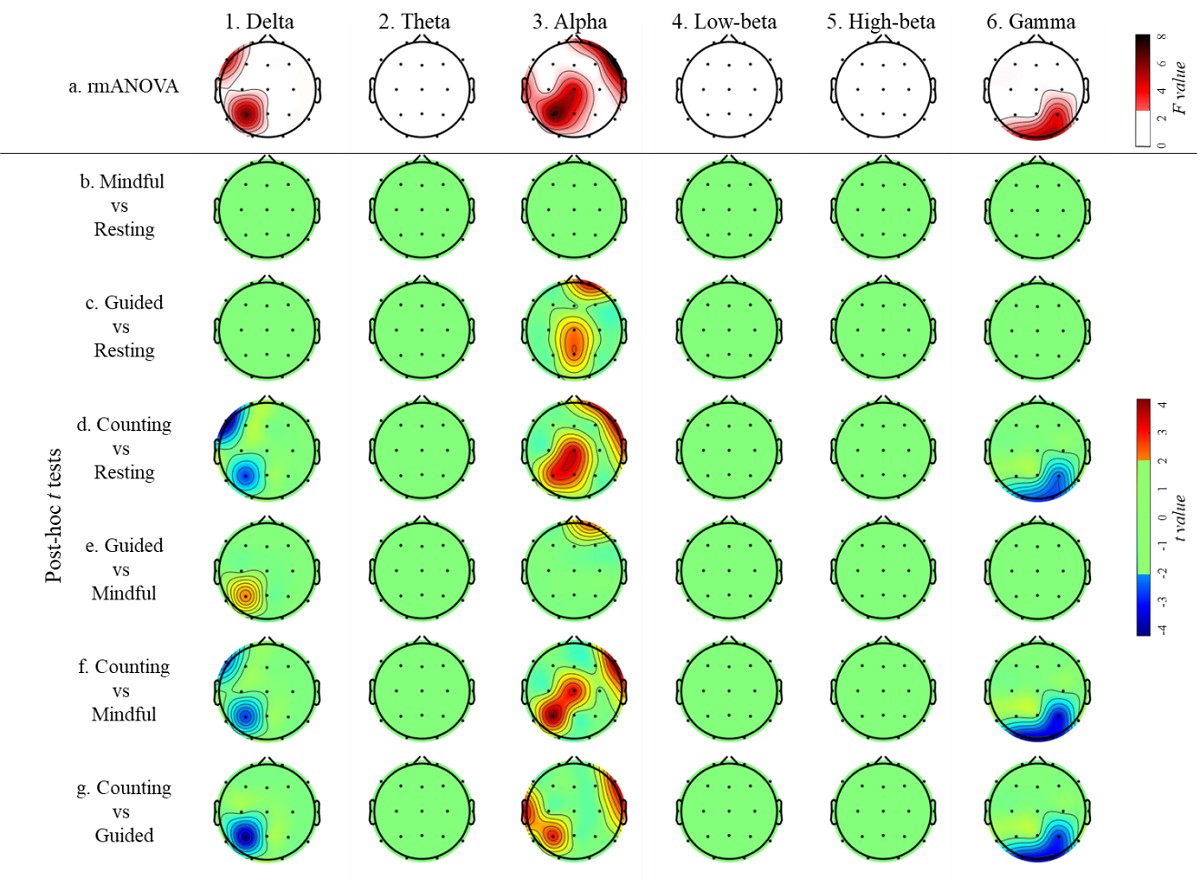
Results of repeated measures ANOVA (rmANOVA) of connectivity outflow across the 4 conditions. False discovery rate (FDR) correction was applied to all the analyses in this figure. All image parts show only the connectivity flow with *P* value with FDR correction (P_FDR_) <0.05, F ≥2.69, and t ≥2.03 or ≤–2.03. In all pairwise comparisons, positive t values represent higher mean values of the former condition. Mindful represents mindful breathing, guided represents guided breathing, and counting represents breath counting.

Figure 3 shows the results of rmANOVA and post-hoc analyses of connectivity outflow among the 4 conditions. There was no connectivity outflow difference in the theta, low-beta, or high-beta bands in any of the scalp regions among the 4 conditions (Figure 3 [parts 2a, 4a, and 5a]; *P* values >.05).

For the delta band, rmANOVA showed significant intercondition differences in the left frontal and left parietal regions (Figure 3 [part 1a]; *P* values <.05). *Mindful breathing* and *guided breathing* showed no connectivity outflow difference when compared with *resting* (Figure 3 [parts 1b and 1c]; *P* values >.05). *Breath counting* led to lower outflow in the left frontal and left parietal regions than all the other conditions (Figure 3 [parts 1d, 1f, and 1g]; *P* values <.05). *Guided breathing* caused higher outflow in the left parietal region than *mindful breathing* (Figure 3 [part 1e]; *P* values <.05).

For the alpha band, rmANOVA showed intercondition differences in the right frontal, bilateral temporal, and left parietal regions (Figure 3 [part 3a]; *P* values <.05). *Mindful breathing* showed no connectivity outflow difference when compared with *resting* (Figure 3 [part 3a]; *P* values >.05). Both *guided breathing* and *breath counting* led to higher outflow than *resting* in the right frontal and central-parietal midline regions (Figure 3 [parts 3c and 3d]; *P* values <.05). *Guided breathing* led to higher outflow than *mindful breathing* in the right frontal region (channel Fp2; Figure 3 [part 3e]; *P* values <.05). *Breath counting* caused higher outflow than *mindful breathing* in the right temporal, central, and left parietal regions (Figure 3 [part 3f]; *P* values <.05), as well as higher outflow than *guided breathing* in the bilateral temporal and left parietal regions (Figure 3 [part 3g]; *P* values <.05).

For the gamma band, rmANOVA showed intercondition differences in the occipital and right parietal regions (Figure 3 [part 6a]; *P* values <.05). *Mindful breathing* and *guided breathing* displayed no connectivity outflow difference when compared with *resting* (Figure 3 [parts 6b and 6c]; *P* values >.05). *Breath counting* led to lower outflow than *resting*, *mindful breathing*, and *guided breathing* in the occipital and right parietal regions (Figure 3 [parts 6d, 6f, and 6g]; *P* values <.05).

### Connectivity Inflow Differences Across the 4 Conditions

**Figure 4:**
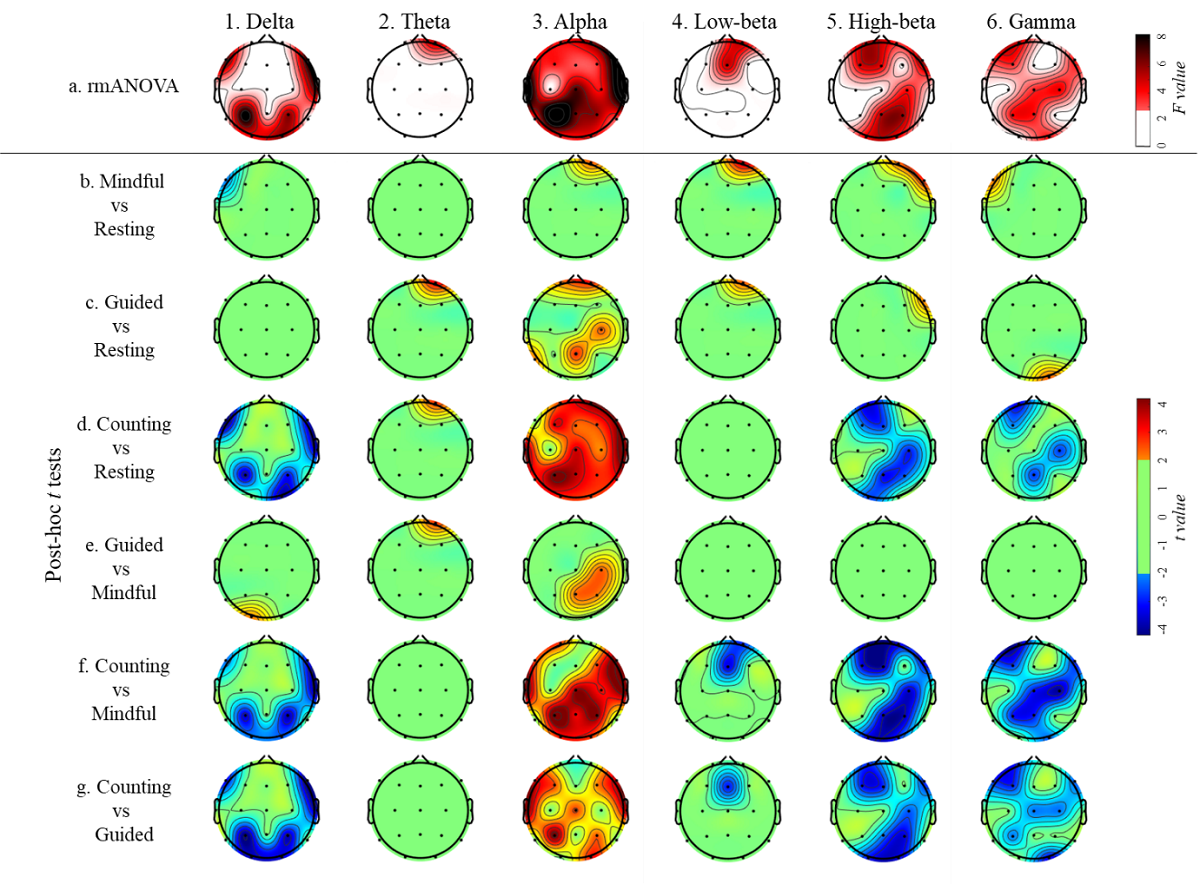
Results of repeated measures ANOVA (rmANOVA) of connectivity inflow across the 4 conditions. False discovery rate (FDR) correction was applied to all the analyses in this figure. All image parts show only the connectivity flow with *P* value with FDR correction (P_FDR_) <0.05, F ≥2.69, and t ≥2.01 or ≤–2.01. In all pairwise comparisons, positive t values represent higher mean values of the former condition. Mindful represents mindful breathing, guided represents guided breathing, and counting represents breath counting.

Figure 4 shows the results of rmANOVA and post-hoc analyses of connectivity inflow among the 4 conditions.

For the delta band, differences in inflow across the 4 conditions were primarily observed in the bilateral frontal, right temporal, bilateral parietal, and occipital regions (Figure 4 [part 1a]; *P* values <.05). *Mindful breathing* exhibited reduced connectivity inflow in the left frontal region compared with *resting* (Figure 4 [part 1b]; *P* values <.05). While *guided breathing* showed no inflow difference relative to *resting* (Figure 4 [part 1b]; *P* values >.05), it did display increased inflow in the left occipital region relative to *mindful breathing* (channel O1; Figure 4 [part 1e]; *P* values <.05). *Breath counting* showed decreased inflow in the bilateral frontal, right temporal, bilateral parietal, and occipital regions relative to *resting*, *mindful breathing*, and *guided breathing* (Figure 4 [parts 1d, 1f, and 1g]; *P* values <.05).

For the theta band, only channel Fp2 showed an intercondition difference in the rmANOVA (Figure 4 [part 2a]; *P* values <.05). There was no inflow difference between the *mindful breathing* and *resting* conditions (Figure 4 [part 2b]; *P* values >.05). Both *guided breathing* and *breath counting* exhibited increased inflow in the right frontal region (channel Fp2) relative to *resting* (Figure 4 [parts 2c and 2d]; *P* values <.05). *Guided breathing* displayed enhanced inflow in the same right frontal region (channel Fp2) relative to *breath counting* (Figure 4 [part 2e]; *P* values <.05). There was no inflow difference between *breath counting* and both *guided breathing* and *mindful breathing* (Figure 4 [parts 2f and 2g]; *P* values >.05).

The alpha band showed differences in inflow across the entire scalp between conditions (Figure 4 [part 3a]; *P* values <.05). *Mindful breathing* led to increased inflow in the right frontal region (channel Fp2) compared with *resting* (Figure 4 [part 3b]; *P* values <.05). *Guided breathing* had a stronger inflow than *resting* in the frontal, left and midline parietal, and right central regions (Figure 4 [part 3c]; *P* values <.05). *Breath counting* exhibited increased inflow globally relative to *resting* (Figure 4 [part 3d]; *P* values <.05). Meanwhile, *guided breathing* showed increased inflow relative to *mindful breathing* in the right central and right parietal regions (Figure 4 [part 3e]; *P* values <.05). When compared with *mindful breathing*, *breath counting* led to greater inflow across all channels, except for Fp2, Fz, F3, and C3 (Figure 4 [part 3f]; *P* values <.05). When compared with *guided breathing*, *breath counting* caused enhanced inflow in the bilateral frontal, bilateral temporal, midline central, bilateral parietal, and right occipital regions (Figure 4 [part 3g]; *P* values <.05).

The rmANOVA revealed an intercondition difference in the low-beta band in the frontal region (Figure 4 [part 4a]; *P* values <.05). Both *mindful breathing* and *guided breathing* exhibited increased inflow compared to *resting*, specifically in the right frontal region (channel Fp2; Figure 4 [parts 4b and 4c]; *P* values <.05). There was no significant inflow difference between *mindful breathing* and *guided breathing* (Figure 4 [part 4e]; *P* values >.05). *Breath counting* showed decreased inflow in the midline frontal region relative to both *mindful breathing* and *guided breathing* (Figure 4 [parts 4f and 4g]; *P* values <.05), but it was comparable to *resting* (Figure 4 [part 4d]; *P* values >.05).

In the high-beta band, both *mindful breathing* and *guided breathing* demonstrated increased inflow in the right frontal region compared with *resting* (Figure 4 [parts 5b and 5c]; *P* values <.05). There was no observed inflow difference between *mindful breathing* and *guided breathing* (Figure 4 [part 5e]; *P* values >.05). *Breath counting* exhibited decreased inflow in the left frontal, right central, midline and right parietal, right temporal, and occipital regions in comparison with the other conditions (Figure 4 [parts 5d, 5f, and 5g]; *P* values <.05).

In the gamma band, *mindful breathing* displayed increased inflow in the left frontal region (channel F7) compared with *resting* (Figure 4 [part 6b]; *P* values <.05). *Guided breathing* showed elevated inflow in the right occipital region (channel O2) relative to *resting* (Figure 4 [part 6c]; *P* values <.05). No inflow difference was observed between *mindful breathing* and *guided breathing* (Figure 4 [part 6e]; *P* values >.05). *Breath counting* revealed decreased inflow in the left frontal, right central, and midline parietal regions relative to *resting* (Figure 4 [part 6d]; *P* values <.05). Furthermore, *breath counting* exhibited diminished inflow in the left frontal, midline and right central, midline and left parietal, and right occipital regions when compared with both *mindful breathing* and *guided breathing* (Figure 4 [parts 6f and 6g]; *P* values <.05).

### Connectivity Flows Among Brain Regions

**Figure 5:**
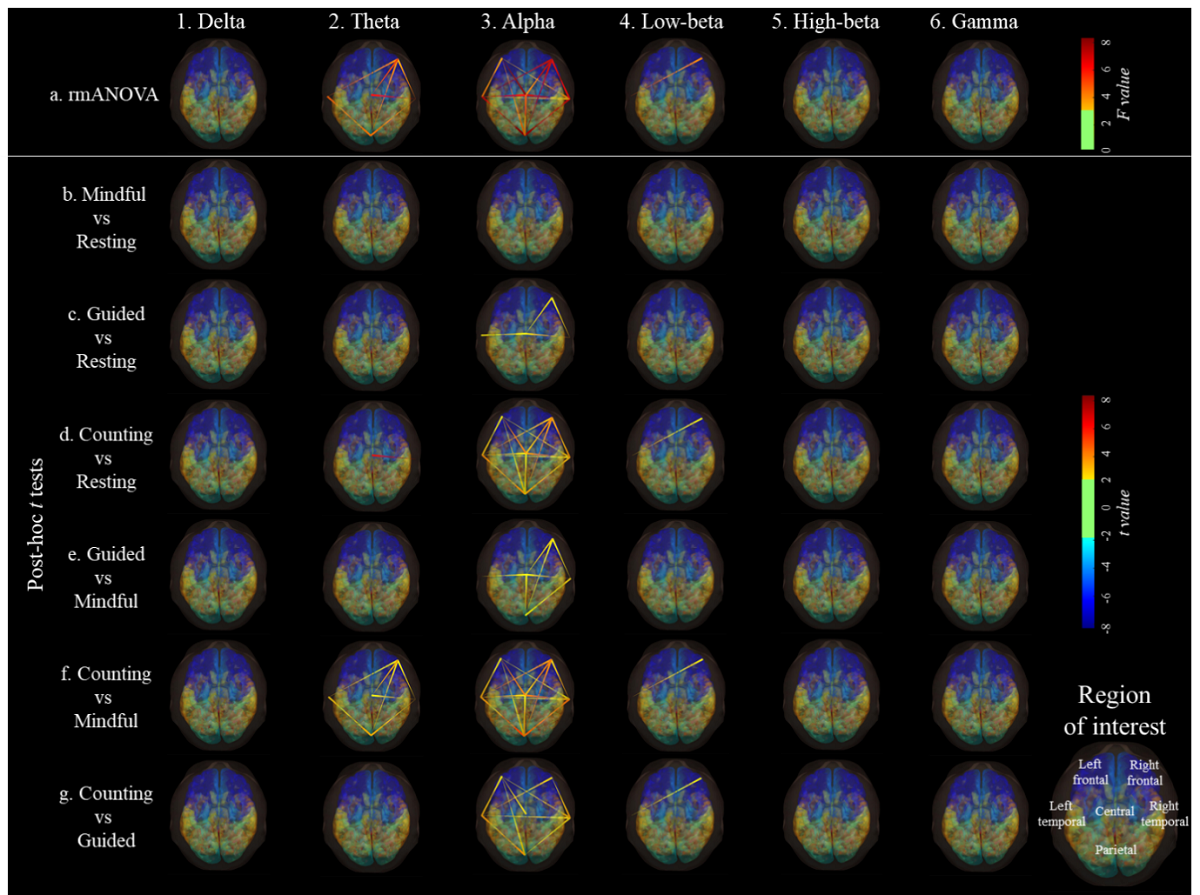
Results of repeated measures ANOVA (rmANOVA) of connectivity flow across 6 brain regions. False discovery rate (FDR) correction was applied to all the analyses in this figure. All image parts show only the connectivity flow with *P* value with FDR correction (P_FDR_) <0.05, F ≥3.10, and t ≥2.03 or ≤2.03. In all pairwise comparisons, positive t values represent higher mean values of the former condition. Mindful represents mindful breathing, guided represents guided breathing, and counting represents breath counting.

Figure 5 shows the results of the rmANOVA and post-hoc analyses examining the connectivity flow among the 6 brain regions.

Notably, the delta, high-beta, and gamma frequency bands did not display any significant differences in connectivity flow across the 6 regions among the 4 experimental conditions (Figure 5 [parts 1a, 5a, and 6a]; *P* values >.05).

For the theta band, compared with *mindful breathing*, *breath counting* led to increased connectivity flow from the right frontal region to the central, parietal, and bilateral temporal regions; from the central region to the right temporal region; between the parietal and bilateral temporal regions; and from the left temporal region to the parietal region (Figure 5 [part 2f]; *P* values <.05).

The alpha band showed robust connectivity differences across conditions (Figure 5 [part 3a]; *P* values <.05). When compared with all the other conditions, *breath counting* showed greater connectivity flow from the right frontal to the central, parietal, and bilateral temporal regions (Figure 5 [parts 3d, 3f, and 3g]; *P* values <.05). *Breath counting* also had greater connectivity flow from the central region to all the other brain regions, as well as from the parietal region to the left frontal, central, and bilateral temporal regions (Figure 5 [parts 3d, 3f, and 3g]; *P* values <.05). *Breath counting* also had greater connectivity flow from the left temporal region to the left frontal and parietal regions, as well as from the right temporal region to the left temporal and parietal regions (Figure 5 [parts 3d, 3f, and 3g]; *P* values <.05). Compared with *resting*, *guided breathing* induced greater connectivity flow from the right frontal region to the central and right temporal regions and from the central region to the bilateral temporal regions (Figure 5 [parts 3d, 3f, and 3g]; *P* values <.05), as well as from the left temporal region to the central region (Figure 5 [part 3c]; *P* values <.05). Compared with *mindful breathing*, *guided breathing* had greater connectivity flow from the right frontal region to the central, parietal, and right temporal regions (Figure 5 [part 3e]; *P* values <.05); from the central region to the bilateral temporal and parietal regions (Figure 5 [part 3e]; *P* values <.05); and from the right temporal region to the parietal region and vice versa (Figure 5 [part 3e]; *P* values <.05)*.* Overall, the right frontal region, but not the left frontal region, was the source of influence for alpha band connectivity in *guided breathing* and *breath counting.*

For the low-beta band, *breath counting* induced greater connectivity flow from the right frontal region to the left temporal region than the other conditions (Figure 5 [parts 3d, 3f, and 3g]; *P* values <.05).

### Questionnaires

We have presented the results and details of questionnaires in Table 3 and Multimedia Appendix 2, respectively. The results suggested that the participants had no extraordinary characteristics. The participants had medium mindfulness levels (FFMQ scores: mean 120.9, SD 15.7), medium sleep quality levels (PSQI scores: mean 5.5, SD 3.2), low depression levels (BDI scores: mean 12.6, SD 8.8), and low anxiety levels (BAI scores: mean 12.6, SD 8.8). As questionnaire responses are not the focus of this paper, we present the data in Table 3 without further interpretation.

**Table 3:** Demographic information and questionnaire results.

Variable				Value (N=51)		Reliability
**Sex, n (%)**						
	Male			26 (51)		—^a^
	Female			25 (49)		—
Age (years), mean (SD)				22.73 (2.15)		—
Years of education, mean (SD)				15.38 (0.96)		—
**Questionnaire (score), mean (SD)**						
	**FFMQ^b^**			120.90 (15.69)		0.853
		Aware	24.24 (4.87)		0.781	
		Description	26.31 (6.90)		0.905	
		Nonjudgmental	19.82 (5.43)		0.805	
		Observation	29.39 (3.69)		0.500	
		Nonreactive	21.14 (4.33)		0.676	
	MoCA^c^			28.71 (1.51)		—
	BDI^d^			12.59 (8.81)		0.900
	BAI^e^			7.41 (6.68)		0.869
	Mind wandering			3.12 (0.98)		0.780
	PSQI^f^			5.49 (3.21)		—
**Breath count per minute, mean (SD)**						
	Resting			14.88 (5.23)		—
	Mindful breathing			13.44 (5.53)		—
	Guided breathing			8.20 (3.34)		—
	Breath counting			12.77 (4.75)		—

^a^Not applicable.

^b^FFMQ: Five Facet Mindfulness Questionnaire.

^c^MoCA: Montreal Cognitive Assessment.

^d^BDI: Beck Depression Inventory.

^e^BAI: Beck Anxiety Inventory.

^f^PSQI: Pittsburgh Sleep Quality Index.

## Discussion

### Principal Findings

Our research questions are whether and how people’s neurological features change when they are performing different breathing training techniques. The rmANOVA results revealed significant differences in EEG band power across the theta, alpha, low-beta, high-beta, and gamma bands over the entire scalp among *resting*, *mindful breathing*, *guided breathing*, and *breath counting* conditions. Outflow analysis identified condition-specific variations in the delta, alpha, and gamma bands, while inflow analysis showed significant variations across all frequency bands. Connectivity flow analyses showed that the right frontal, central, and occipital brain regions were predominantly influential in *guided breathing* and *breath counting*. Notably, in all the analyses, *guided breathing* and *breath counting* exhibited distinguishable EEG features compared to *resting.* In contrast, the EEG features of *mindful breathing* did not significantly differ from those of *resting*.

### Comparison With Prior Work

#### Internal and External Attention Nature of VR-Guided Breathing Training

Our results of *breath counting* are consistent with the EEG evidence found in previous breathing training studies that this technique increases alpha band power globally relative to baseline [49,50,52,95]. Our results of *breath counting* and *guided breathing* also agree with previous findings showing decreased beta power [48] and gamma power [95]. In contrast, *guided breathing* showed decreased alpha band power relative to baseline, suggesting that *guided breathing* and *breath counting* may have different neurological mechanisms. We suggest that the underlying mechanism is internal-external attention focus.

Internal attention focuses on internally generated information, whereas external attention focuses on sensory inputs [96]. Breathing training or meditation is usually considered as a process of internal attention, but VR-based breathing training is somewhat an external attention process [97]. Participants in our study, for example, were inevitably influenced by external cues as they were instructed to open their eyes and were exposed to the VR swaying meadow. We observed 2 comparable phenomena in our study. First, we found people who were executing internal attention tasks to have increased alpha band power globally [98], especially in the parietal region [99], which is consistent with our results of *breath counting* (see Figure 2 [part 3d]). Second, we found people who were conducting external attention tasks to have robustly decreased alpha band power in the frontal-central region [100], which is consistent with our results of *guided breathing* (see Figure 2 [part 3c]).

An explanation for this observation is that *breath counting* is more likely to involve internal attention processing, whereas *guided breathing* is more likely to involve external attention processing. In the *breath counting* condition, the participants were instructed to internally count their breath cycles; thus, external interference was less salient. In the *guided breathing* condition, however, the participants were instructed to breathe at a fixed tempo with a swaying meadow as the visual cue, and therefore, external interference was essential. External- and internal-oriented tasks recruited different neurological networks: functional magnetic resonance imaging (fMRI) evidence showed that external attention recruited the executive-related network, including the visual cortex, inferior parietal lobule, thalamus, and primary motor area, while internal attention recruited the somatosensory-related network, including the medial and inferior parietal region, insula, posterior cingulate, hippocampus, and lingual gyrus [101-103]. As those fMRI connectivity results showed that both attention pathways crossed at the anterior insula cortex, the researchers proposed that the 2 pathways may be intertwined, forming an integrated cognition to fine-tune the delicate inner sensation [101,104]. It is likely that breathing techniques cannot be solely internal or external, but they can have pathway preferences [105]. This argument also explains why both breathing techniques can be beneficial for practitioners.

However, *mindful breathing* did not show robust EEG power change relative to baseline. This finding aligns with previous mindfulness studies, where a pronounced alteration in high-frequency EEG power due to mindful breathing was observed only after an 8-week mindfulness training, not preceding it [54,57]. Although Weng et al [97] advocated the internal nature of breathing training, our results did not provide a clear indication of whether mindful breathing should be internally or externally attention oriented. Therefore, we predict that *mindful breathing* should be independent of *breath counting* and *guided breathing*. Our EEG evidence suggests that using *breath counting* or *guided breathing* instructions may not yield the desired mindfulness outcomes.

Despite the ongoing debate regarding whether breathing training should be internally or externally oriented, 2 evidence-based studies have suggested that VR-based training is effective at lowering emotional distress indexes [3,64] and chronic pain regardless of the breathing technique being followed [106]. We speculate that internal attention is not necessary for practitioners to benefit from breathing training. In contrast, external attention could provide beginners, who require explicit breath guidance, with easier initiation, and this theory is in accordance with the design of breathing training applications [42,43]. To conclude, external and internal attention-oriented breathing training can benefit mental health, and practitioners may choose whichever technique suits them.

#### Connectivity Results Suggest a Link Between Attention Network and Breathing Training

According to our effective connectivity results, the alpha band showed the most robust outflow difference in the right frontal and left parietal regions among the 4 conditions, with the outflow decreasing in the order of *breath counting*, *guided breathing*, *mindful breathing*, and *resting* (see Figure 3 [parts 3b, c, d, e, f, and g]). Our connectivity flow analyses also showed a similar phenomenon (see Figure 5 [parts 3b, c, d, e, f, and g]). Two theories can plausibly explain this phenomenon.

One theory accounting for EEG evidence on cognitive resources suggests that the increased bidirectional coordination between the frontal and visual regions corresponds to the effort made by people to withstand visual interference when executing internal attention tasks [107]. Our results of increased frontal-parietal connectivity represent the participants’ attempts to resist external interferences and focus on the internal breathing task. This argument follows the thought that breathing training is an internal attention process [97], and internal and external attention can be considered a pair of antagonistic processes. This argument, however, does not address the possibility that internal and external attention may cooperate in breathing [105].

Another theory of the attentional network based on fMRI evidence suggests that the dorsal attention network (DAN) is recruited when people selectively attend to external stimuli [108]. The DAN comprises the frontal eye field (at the frontal lobe) and the intraparietal sulcus (at the parietal lobe), which were also revealed to be the major connectivity outflow regions. The DAN connectivity reflects the level of integration between external cues and mental representations and the ability to switch between internal and external attention [109]. Considering the existence of internal-external attentional cooperation in breathing [105], the increased connectivity between the frontal and parietal regions (which overlaps with the DAN) may suggest a refined attentional cooperation in breathing training.

Breathing training can also be beneficial to people with developmental disorders [9,10,110]. Studies on autism further supported the DAN argument, reporting that patients with autism commonly have decreased global alpha connectivity [111,112]. An EEG source reconstruction study found decreased alpha band functional connectivity in DAN in patients with autism, and this phenomenon was thought to correspond to the difficulty in switching between externally and internally oriented thoughts [112]. That is, the level of DAN connectivity is likely to reflect the ability of external and internal attention to cooperate. The alpha connectivity level is related to attention power in autism [112], and this partially explains why we found robust connectivity results in the alpha band. Nevertheless, we can only make a discreet inference here, as our EEG evidence does not directly shed light on DAN.

### Strengths of This Study

#### Breath Signals and EEG Features as Biomarkers of Breathing Training

Breath signals and EEG features can also serve as BF indicators in breathing training. Breath signals have been widely employed as BF indicators in breathing training [4], and this study further validated the potential of EEG features to be used as biomarkers. EEG power and power ratio are commonly used as BF biomarkers because they are computationally affordable [13]. Our results also suggest that alpha band effective connectivity can differentiate various breathing training techniques. Breathing training instructors may use EEG signals to monitor the progress of trainees.

According to our findings, the alpha, beta, and gamma band powers are ideal EEG markers to identify whether *guided breathing* and *breath counting* are correctly performed. Our findings agree with those of some BF psychophysiological intervention studies in that the alpha power [113], beta power [114], and bilateral alpha power ratio [115,116] are particularly useful as EEG BF markers. Our findings further suggest that breathing training instructors should monitor globally increased alpha power relative to baseline if they are administering *breath counting* training but globally decreased beta or gamma power if they are administering *guided breathing* training. However, although previous BF psychophysiological intervention studies have usually considered theta power as a BF marker [114,117], our findings do not support the use of this EEG marker, given that we found only a limited theta power difference in the occipital region. Overall, alpha, beta, and gamma powers were more promising than theta power as EEG markers.

Our findings also suggest that different EEG markers must be monitored for *mindful breathing* relative to *guided breathing* and *breath counting*. Our findings showed that first-time *mindful breathing* practitioners would show no significant difference in EEG markers compared with baseline. Finding a robust alpha, beta, or gamma power change in novice *mindful breathing* practitioners would indicate that the breathing training technique was not correctly performed. As mentioned, high-frequency EEG power changes would only occur after mindfulness training [54,57]. Thus, mindfulness instructors may monitor practitioners’ high-frequency EEG power change for evaluation.

#### Use of VR-BR Breathing Training In-House and in Clinical Practice

To recreate the environment of an in-house breathing training session of a common person, this study implemented a common in-house setting as much as possible. Our laboratory, in which experimental sessions took place, is a common room without a soundproof design. Participants could hear the sound of an air conditioner and the echo produced by their own actions. This experiment should be repeatable in any other room with VR-BF devices. Common breathing training learners probably have no soundproof room, and they actually do not need one when they have VR devices. An advantage of VR-BF breathing training is that the immersive 360° visual environment of VR also isolates users from environmental distractions, regardless of visual or acoustic distributions [24]. People of this modern society are always challenged by the distractions contesting for their attention, and people with limited or no training are vulnerable to that [118]. For beginners of breathing training, VR devices isolate them from distractions and give them a digital object to focus on, and focusing on a designated digital target should be close to people’s daily experience [3,26]. This artificial environment may equip breathing training beginners with basic attentional training before they start practicing breathing training in the traditional way. An easier initiation should reduce the dropout rate of self-help breathing training and eventually improve trainees’ mental health.

Although previous work on VR-BF self-help breathing training showed positive outcomes in trainees, we do not suggest that self-help breathing training can replace professional instructors. In fact, instructors and VR-BF technologies may cooperate. With VR-BF technologies, instructors can accurately and simultaneously monitor trainees’ situations and provide additional guidance whenever necessary. Some clinical therapists attempted to provide online breathing training programs since the COVID-19 pandemic started [119-122]. Traditional vision-based online training programs have a shortcoming that therapists can only acquire limited information on trainees, as cameras usually show only the front of them [122]. With VR-BF technologies, therapists can monitor trainees’ physiological situations as BF data can be monitored by therapists through the internet. Using the internet and VR-BF technologies, therapists may popularize their training programs to whoever is in need without sacrificing too much training quality [123,124].

#### Response to the COVID-19 Pandemic

The COVID-19 pandemic has been a global crisis. Luckily, the experiments of this study were conducted in Taiwan. Taiwan had no lockdown policy, and therefore, laboratory regulations and our data were unaffected. On the other hand, owing to epidemic control and city lockdowns, the public around the world generally had very limited medical access, and their mental and physical health faced unprecedented challenges [119,120]. In-house VR-BF self-help breathing training can at least be a supplement to people’s need of mental health care under the given circumstance. Self-help breathing training has no risk of interpersonal exposure, and therefore, it was one of the few acceptable health care solutions available to the public. 

Another advantage of VR-BF self-help breathing training is that it is a cost-effective health care solution. Professional mental health attention or breathing training programs usually have a considerable cost. On the other hand, the hardware of VR-BF self-help breathing training is relatively affordable to most people. The HTC Vive VR headset that we used in this study costs about US $560 and can be purchased online [125]. Although we employed a respiration belt to collect participants’ breath signals in our study, smartphones can be a replacement to detect people’s breathing rate [41]. Once the VR-BF self-help breathing training equipment is purchased, breathing training learners should have very limited expenses afterward. The cost-effective feature of VR-BF self-help breathing training should especially benefit the grassroots population and whoever is concerned about the cost of breathing training courses.

### Limitations

There were some limitations. First, because of the cross-sectional nature of this study, electrophysiological changes occurring during long-term breathing training could not be evaluated. Future investigations are essential to delve into the behavioral and neural transformations, and the underlying mechanisms induced by different VR-guided breathing training styles. This will contribute to a more comprehensive understanding of the long-term impacts and benefits of these interventions.

Second, as this study focused on novice mindful breathing practitioners, the evidence may not address the situation of how experienced breathing training practitioners would neurologically respond to VR-BF integrated training. It is also unclear whether VR-BF integrated technologies would make any difference (good or bad) to the training efficacy of experienced breathing training practitioners. Researchers may focus on experienced breathing training practitioners in terms of VR-BF integrated training.

### Future Directions

Although the hardware of VR-BF self-help breathing training is relatively affordable, a missing piece for the popularization of such breathing training is the corresponding software. There is a need for an open-access breathing training application that integrates different models of VR headsets and BF devices (eg, smartphones, respiration belts, and consumer-level EEG headsets). This need is unaddressed. Subsequent studies may focus on the development of a VR-BF self-help breathing training application.

This study provides a list of electrophysiological features (ie, band power and effective connectivity) of different breathing training techniques. However, the long-term neurological mechanism changes due to different breathing training techniques are mostly unaddressed. Manufacturers can plausibly integrate their consumer-level EEG headsets with a breathing training application to monitor trainees’ electrophysiological signal changes. Such a breathing training program with EEG BF may then introduce the designated breathing training effect to trainees. We do not want to make any premature statement as the long-term electrophysiological features are yet to be discovered. Further studies on the EEG features of practicing different breathing training techniques for a longer period are needed for the commercialization of breathing training applications with EEG BF.

This study analyzed the data of 51 participants, and we believe that this size was sufficient to produce stable and reliable results. Previous empirical studies on VR-BF and breathing training usually had 9 to 45 participants [1,3,30,33], and the sample size of this study was relatively large in this area of research. To strengthen our analysis, we conducted FDR correction in all assessments [94]. Nevertheless, further studies with a larger sample size to examine the conclusion of this study are encouraged.

### Conclusions

This preliminary study attempted to elaborate on the electrophysiological differences among different breathing training styles. Our results suggest that *mindful breathing*, *guided breathing*, and *breath counting* could be effectively administered through integrated VR and BF breathing training. We also observed the differences in EEG features among these breathing styles, particularly in band power and effective connectivity. These EEG features hold promise as potential biomarkers in integrated VR and BF breathing training for monitoring and assessing the progress of practitioners.

## Appendix

Multimedia Appendix 1 Demonstration of synchronized data collection.

Multimedia Appendix 2 Details of the questionnaire package used in this study.

Multimedia Appendix 3 CONSORT-EHEALTH (V 1.6.1) checklist.

## Abbreviations

BAI: Beck Anxiety Inventory
BDI: Beck Depression Inventory
BF: biofeedback
DAN: dorsal attention network
dDTF: direct directed transfer function
ECG: electrocardiography
EEG: electroencephalography
FDR: false discovery rate
fMRI: functional magnetic resonance imaging
MoCA: Montreal Cognitive Assessment
PSQI: Pittsburgh Sleep Quality Index
rmANOVA: repeated measures ANOVA
VAR: vector autoregressive
VR: virtual reality

## Data Availability

Following the regulations of the Taiwan authority and the institutional review board, the data collected for this study are available only for the intended research topic and to the corresponding personnel.
