# Supplementary material for: Neurological Evidence of Diverse Self-Help Breathing Training With Virtual Reality and Biofeedback Assistance: Extensive Exploration Study of Electroencephalography Markers
Source: JMIR Form Res. 2024 Dec 6;8:e55478. doi: 10.2196/55478 (PMC11662191; doi:10.2196/55478)
Supplement: Multimedia Appendix 1 [file formative_v8i1e55478_app1.docx]

**Multimedia Appendix 1.** Demonstration of synchronized data collection. (a) EEG, (b) ECG, and (c) breathing.

**
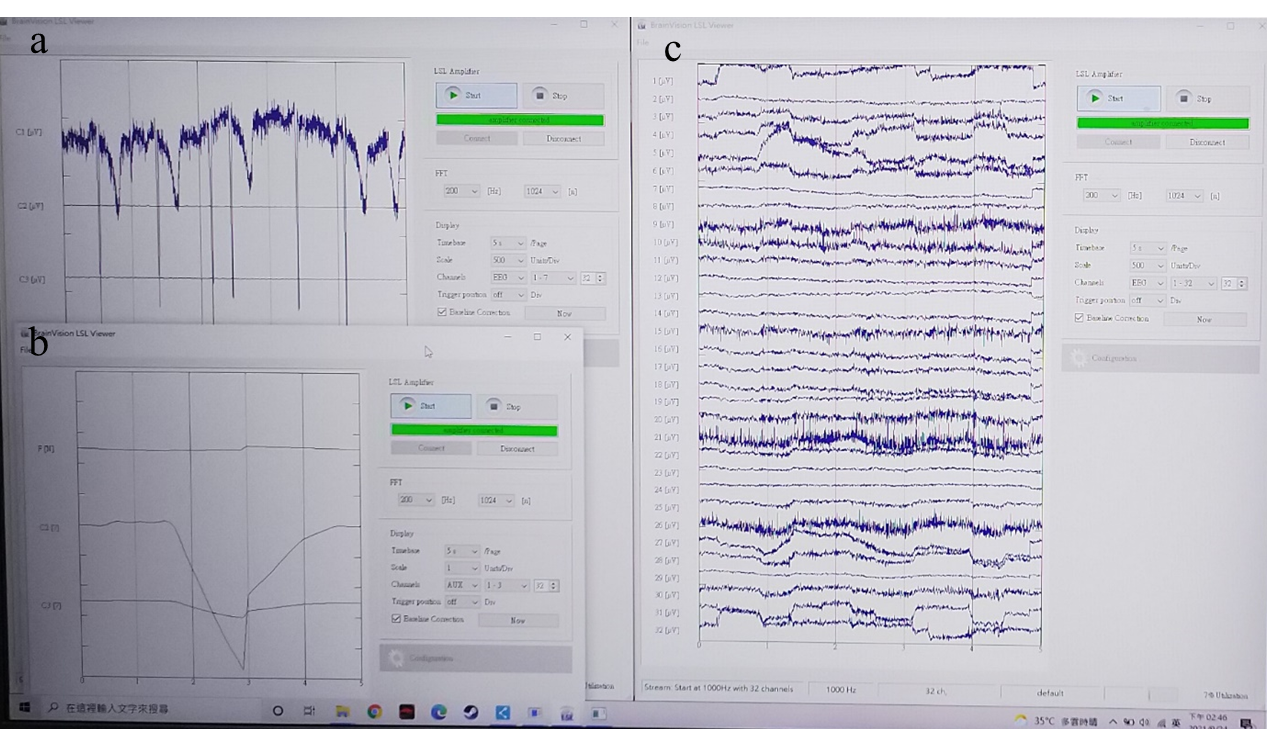
**
