# Supplementary material for: Neurological Evidence of Diverse Self-Help Breathing Training With Virtual Reality and Biofeedback Assistance: Extensive Exploration Study of Electroencephalography Markers
Source: JMIR Form Res. 2024 Dec 6;8:e55478. doi: 10.2196/55478 (PMC11662191; doi:10.2196/55478)
Supplement: Multimedia Appendix 2 [file formative_v8i1e55478_app2.docx]

**Multimedia Appendix 2.** Details of the questionnaire package used in this study.

The Five Facet Mindfulness Questionnaire (FFMQ) measures people’s mindfulness level using 39 items [1,2]. It is composed of five subscales that depict different aspects of mindfulness, namely awareness, describing, observing, non-judging, and non-reactiveness. All items are scored from 1 (never or very rarely true) to 5 (very often or always true), with the total score ranging from 39 to 195. A higher score represents greater mindfulness. The reliability of the FFMQ is good, with a Cronbach’s α of .85.

The Montreal Cognitive Assessment (MoCA) is a brief clinical screening questionnaire that detects mild cognitive impairment [3]. It includes 30 points and can be administered in 10 minutes. The MoCA assesses short-term memory, executive functions, attention, visuospatial ability, phonemic fluency, verbal abstraction, and time and place orientation. Nasreddine, Phillips, Bedirian, Charbonneau, Whitehead, Collin, Cummings and Chertkow [3] suggested MoCA scores of 25 or lower indicate mild cognitive impairment.

The Beck Depression Inventory (BDI) is a classical self-reported questionnaire that measures the depression level [4]. It comprises 21 items that are scored from 0 to 3, with the total score ranging from 0 to 63. A higher score represents greater trait depression. The reliability of the BDI is very high, with a Cronbach’s α of .90.

The Beck Anxiety Inventory (BAI) is a self-reported questionnaire that measures the anxiety level [5]. It comprises 21 items that are rated from 0 (no concern) to 3 (high level of concern), with the total score ranging from 0 to 63. A higher score represents greater trait anxiety. The reliability of the BAI is high, with a Cronbach’s α of .86.

The Mind Wandering: Spontaneous (MW-S) scale is a self-reported measure of unintentional mind wandering [6]. It has four items that are rated from 1 (rarely) to 5 (always). A higher score represents greater unintentional mind wandering trait. The reliability of the MW-S scale is acceptable, with a Cronbach’s α of .78.

The Pittsburgh sleep Quality Index (PSQI) is a self-reported questionnaire that assesses sleep quality [7]. The PSQI measures multiple aspects of sleep, including the duration of actual sleep, the frequency of and concern over sleep-interrupting incidents, and the use of sleeping pills. According the scoring rules of [7], the responses to different items are converted and computed into a sleep quality index.

**References**

1. Baer RA, Smith GT, Lykins E, et al. Construct validity of the five facet mindfulness questionnaire in meditating and nonmeditating samples. *Assessment*. 2008/09// 2008;15(3):329-342. doi:10.1177/1073191107313003

2. Huang FY, Wu CW, Bhikshu HM, Shih GH, Chao YP, Dai CT. Validation of the Taiwanese version of the five facet mindfulness questionnaire (T-FFMQ). *Psychological Testing*. 2015 2015;62(3):231-260.

3. Nasreddine ZS, Phillips NA, Bedirian V, et al. The Montreal Cognitive Assessment, MoCA: A brief screening tool for mild cognitive impairment. *J. Am. Geriatr. Soc.* Apr 2005;53(4):695-9. doi:10.1111/j.1532-5415.2005.53221.x

4. Beck AT, Steer RA, Carbin MG. Psychometric properties of the Beck Depression Inventory: Twenty-five years of evaluation. *Clin. Psychol. Rev.* 1988;8(1):77-100. doi:10.1016/0272-7358(88)90050-5

5. Beck AT, Epstein N, Brown G, Steer RA. An inventory for measuring clinical anxiety: Psychometric properties. *J. Consult. Clin. Psychol.* 1988;56(6):893.

6. Carriere JSA, Seli P, Smilek D. Wandering in both mind and body: individual differences in mind wandering and inattention predict fidgeting. *Can. J. Exp. Psychol.* Mar 2013;67(1):19-31. doi:10.1037/a0031438

7. Buysse DJ, Reynolds CF, 3rd, Monk TH, Berman SR, Kupfer DJ. The Pittsburgh Sleep Quality Index: A new instrument for psychiatric practice and research. *Psychiatry Res.* May 1989;28(2):193-213. doi:10.1016/0165-1781(89)90047-4
